# Psychometric Properties of a Risk Tool Across Indigenous Māori and European Samples in Aotearoa New Zealand: Measurement Invariance, Discrimination, and Calibration for Predicting Criminal Recidivism

**DOI:** 10.1177/10731911231153838

**Published:** 2023-02-22

**Authors:** Darcy J. Coulter, Caleb D. Lloyd, Ralph C. Serin

**Affiliations:** 1 Centre for Forensic Behavioural Science, Swinburne University of Technology and Forensicare, Alphington, Victoria, Australia; 2 Carleton University, Ottawa, Ontario, Canada

**Keywords:** DRAOR, Indigenous, measurement invariance, recidivism, calibration

## Abstract

Due to recent legal cases highlighting a lack of cross-ethnicity validity research using correctional risk assessment tools, we evaluated psychometric properties of Dynamic Risk Assessment for Offender Re-entry (DRAOR) scores across Māori (*n* = 1,812) and New Zealand (NZ) European samples (*n* = 1,211) in Aotearoa NZ. Using routine administrative data, our analyses suggested scoring properties were invariant across ethnicity for 15 of 19 items. Discrimination properties were also equivalent, but we observed a higher recidivism base rate among Māori participants, consistent with official statistics. Consequently, calibration analyses using a fixed follow-up (*N* = 372) demonstrated higher predicted recidivism rates for Māori participants at each DRAOR score. This suggests that Māori participants with similar levels of DRAOR-assessed need factors as NZ European participants experienced relatively greater continued justice contact. DRAOR users should prioritize delivering quality case management to clients, recognizing that both case-specific and systemic factors may underlie differential base rates.

The Indigenous^1^ Peoples of Aotearoa New Zealand (NZ) (Māori), Australia (Aboriginal and Torres Strait Islander peoples), and Canada (First Nations Peoples, Inuit, and Métis) are substantially overrepresented in their respective country’s prison populations (Australian Law Reform Commission, 2017; Crichton et al., 2015; Malakieh, 2019). Although distinct in most aspects, the Indigenous Peoples of Australia, Canada, and Aotearoa NZ have experienced and continue to experience similar injustices that arise from colonial histories and perpetuate correctional overrepresentation. These core factors include disadvantages such as socioeconomic marginalization, dismissal of Indigenous knowledge when developing justice structures and practices, and conscious actions of governments such as the forced removal of Indigenous children from their families (Cunneen & Tauri, 2019). Tamatea (2017) acknowledged that the current best practice in correctional assessment and rehabilitation does not align well with the worldviews of Indigenous peoples, and Cunneen and Tauri (2019) argued that Indigenous peoples will continue to be overrepresented in corrections until governments and academics support Indigenous peoples’ desire for self-determination. Arguably, one necessary (but insufficient) element that can support self-determination is developing an understanding of practices that may create, sustain, or fail to address inequalities in corrections, such as risk assessment. Tamatea (2017) specifically called for researchers to more proactively reckon with the needs of Indigenous peoples by reexamining risk assessment practices.

Researchers in corrections often develop and validate risk assessment tools with predominantly non-Indigenous samples but then use these tools to assess the risk of recidivism for Indigenous peoples without explicit reevaluation (Olver et al., 2014; J. P. Singh et al., 2011). Recent legal hearings in both Canada (*Ewert v. Canada*, 2015, 2018) and Australia (*Director of Public Prosecutions for Western Australia v. Samson*, 2014) have highlighted that this practice is inappropriate. The Supreme Court of Western Australia questioned the admissibility of the Static-99 (Hanson & Thornton, 1999) with Indigenous Australian males and concluded that due to a lack of validation with Indigenous samples, the Static-99 was not valid with Indigenous people and deserved little weight (*Director of Public Prosecutions for Western Australia v. Samson*, 2014). Similarly, in Canada, *Ewert v. Canada* (2015) ruled there was insufficient psychometric evidence to support the federal corrections service using numerous Canadian-developed, internationally popular, risk tools with Indigenous Canadian persons. Later, *Canada v. Ewert* (2016) overturned this decision, stating that lack of evidence did not disallow using these tools with Indigenous persons, but later still, the Supreme Court of Canada decided in a split decision that Canada’s federal corrections service had failed in their obligation to validate these tools’ psychometric properties within Indigenous populations, even if it was not unreasonable to use them (*Ewert v. Canada*, 2018).

When businesses or governments make case-based decisions that affect people’s lives, there are clear advantages for people from marginalized groups if decision criteria are well-defined, transparent, and standardized. Compared with allowing decision criteria to remain unspecified, obscure, and applied arbitrarily to each individual, it is much preferable to develop decision proformas that can be explicitly evaluated to identify (and correct for) sources of bias. If expecting systemic bias that is simultaneously broader than and actioned through individual decision-makers, it is incongruent to rely solely on decision-maker goodwill, adopt a “black box” human decision-making approach, and expect that staff training is sufficient to fully remove bias, especially if hypothesizing that underlying biases are implicit (i.e., outside of conscious awareness). In other words, evaluation of a structured proforma is the key mechanism for identifying and quantifying bias to increase transparency. Unstructured approaches are difficult to investigate and possibly impossible to correct, yet structured criteria are not inherently unbiased simply because they are structured; algorithms and the data used to create them are generated by human processes that may introduce bias at each stage, from data creation to item selection to scoring behavior (Eckhouse et al., 2019). Evaluations, especially ecologically valid evaluations, are essential.

For risk assessment, when researchers conduct evaluations of routine practice (i.e., field studies) outside of controlled research environments, they measure a risk tool’s actual performance rather than potential performance (Edens & Boccaccini, 2017). Although field studies cannot prioritize scoring consistency and interrater reliability, findings are more generalizable to other applied settings and the tool’s intended end users. Real-world evaluation contexts may be particularly susceptible to examiner biases, making field studies an important opportunity to test for cross-cultural differences (see Edens & Boccaccini, 2017). Therefore, in this study, using data collected within routine practice, we evaluated measurement invariance, predictive discrimination, and predictive calibration properties within data collected through a structured case management tool used nationally in Aotearoa NZ among both NZ Māori and NZ European people.

## Correctional Assessment With Indigenous Peoples Outside Aotearoa NZ

Shepherd and Lewis-Fernandez (2016) provided a comprehensive overview of the problems that can likely arise when assessing Indigenous peoples’ recidivism risk with tools developed and validated with predominantly White samples. Indigenous peoples of Canada and Australia typically receive higher scores on correctional risk assessments compared with White samples (Babchishin et al., 2012; Muir et al., 2020; Olver, Sowden et al., 2018; Perley-Robertson et al., 2019; Stockdale et al., 2014; Wilson & Gutierrez, 2014; Wormith et al., 2015) and lower scores on strength domains (Lovatt et al., 2022; Muir et al., 2020; Shepherd et al., 2014). However, observing different group averages can be, on its own, a misleading test and researchers need to further evaluate the discrimination and calibration properties of risk tools across subgroups (Lee & Hanson, 2017; see L. M. Helmus & Babchishin, 2017, for a fuller discussion of these psychometric properties).

Discrimination refers to whether risk scores differentiate recidivists and nonrecidivists such that higher risk scores are associated with a greater likelihood of recidivism compared with lower risk scores. Using statistics that quantify decision-making classification errors (i.e., false positives), studies generally demonstrated poorer discrimination within Indigenous subsamples compared with White subsamples in Canada and Australia (Olver, Sowden et al., 2018; Shepherd et al., 2015; Spiranovic, 2012; Thompson & McGrath, 2012; Wilson & Gutierrez, 2014; Wormith et al., 2015). Among Canadian and Australian youth, studies measuring strength factors generally found weaker discrimination among Indigenous subsamples compared with White subsamples (Lovatt et al., 2022; Muir et al., 2020; Shepherd et al., 2014). Findings were inconsistent depending on the sample and the outcome, such that one study demonstrated equivalent discrimination for nonviolent recidivism only (Shepherd et al., 2014) and another demonstrated stronger discrimination for violent recidivism among Indigenous youth, yet no discrimination for nonviolent recidivism (Lovatt et al., 2022). In a meta-analysis, Babchishin et al. (2012) also demonstrated poorer discrimination using the 2002 update to Static-99 for Indigenous than non-Indigenous samples, but similar levels of discrimination using Static-99 and Static-99R (L. Helmus et al., 2012). Furthermore, in a large meta-analysis of the Level of Service scales (e.g., scales stemming from the Level of Service Inventory; Andrews, 1982), Olver et al. (2014) found no differences in their discrimination of Indigenous and nonminority Canadians in terms of general recidivism. Researchers often use areas under the curve (AUC) statistics to estimate discrimination, but various other statistics (e.g., hazard ratios or odds ratios) also measure discrimination.

Calibration refers to how well the observed (or model estimated) recidivism rates for a subgroup are aligned with known rates of the tool’s normative sample at each score on the tool. However, when a risk tool does not have a normative sample, the definition of calibration can be extended to evaluate recidivism rate consistency across two subsamples. There are few calibration tests using data from Indigenous samples. However, Olver, Neumann, and colleagues (2018) reported higher general and violent recidivism rates at most Psychopathy Checklist–Revised (PCL-R; Hare, 1991, 2003) scores among Canadian Indigenous participants compared with non-Indigenous participants. Similarly, Olver, Sowden, and colleagues (2018) reported relatively higher violent recidivism rates among Canadian Indigenous participants using Violence Risk Scale–Sexual Offense version (VRS-SO; Wong et al., 2003) scores. For sexual recidivism, the VRS-SO estimated comparatively higher rates among lower scale scores, yet comparatively lower rates among higher scale scores, compared with non-Indigenous participants. Furthermore, Canadian Indigenous people with lower range scores on the Ontario version of the Level of Service Inventory (LSI-OR; Andrews et al., 1995) had higher estimated recidivism rates than similarly low-scoring non-Indigenous people (Wilson & Gutierrez, 2014). Whereas high-risk Canadian Indigenous samples demonstrated predicted sexual recidivism rates consistent with the Static-99R/Static-2002R (L. Helmus et al., 2012) norms, comparable White samples demonstrated recidivism rates significantly lower than the norms (Lee et al., 2020).

When two subgroups have similar average recidivism base rates, researchers can design tools that simultaneously achieve similar degrees of both classification accuracy (i.e., discrimination) and score calibration across groups; however, when base rates differ, it is known property that both features cannot simultaneously be equal across groups (Berk et al., 2021; Eckhouse et al., 2019; Kleinberg et al., 2016). This inherent trade-off means that when researchers and agency partners collaborate to design and implement tools, they must (a) be aware when important subgroups in their assessed population have different recidivism base rates and (b) consider the implications of choosing to prioritize either discrimination or calibration properties, which may depend on a tool’s practical purpose. Not all agencies assess and/or report recidivism base rates by subpopulation. However, NZ Department of Corrections helpfully publishes annual reports that, for example, specified that between 2010 and 2020, the 2-year postprison reconviction rates were, on average, 11.4% higher among Māori than NZ European people, ranging from 9.3% (NZ Department of Corrections, 2011) to 15.9% higher (NZ Department of Corrections, 2018). Thus, when scoring rules are consistent for all people, these unequal subgroup base rates guarantee any applied risk tool cannot demonstrate both equal classification and calibration accuracy across subgroups simultaneously.

The primary purpose of risk tools is to predict recidivism through accurate classification and/or calibration. Even when a tool’s purpose is to assess rehabilitation needs, items logically must be associated with recidivism (Bonta & Andrews, 2017). Yet, a further potential source of inequality occurs when raters systematically score items differently for different subgroups, such that scores measure theoretically different latent constructs (Putnick & Bornstein, 2016). We are aware of a limited number of studies that have assessed measurement invariance across Indigenous and White samples for forensic tools or risk tools, with most using the PCL-R family of measures of psychopathy across Canadian Indigenous and White samples (McCuish et al., 2018; Olver et al., 2013; Olver, Neumann et al., 2018). These studies demonstrated measurement invariance, both in terms of equality of correlations between tool items and their underlying latent constructs (metric invariance) and equality of item thresholds, such that subgroups share the same point on the underlying latent construct at which a rating progresses to the next score (scalar invariance; see Mewton et al., 2016). McCuish et al. (2018) concluded that the PCL-R be used as part of a broader package of risk and protective assessment tools with Indigenous Peoples. By contrast, Huang and colleagues (2021) used an alternative analytical method and concluded that Canadian Indigenous and White youth differed in likelihood of item endorsement on two subcomponents (Education and Substance Abuse) of the Youth Level of Service/Case Management Inventory (YLS/CMI; Hoge & Andrews, 2002).

## Aotearoa NZ Context

For decades, scholars have discussed overrepresentation of Māori in Aotearoa NZ’s criminal justice system (see Jackson, 1988), focusing explanations on the impacts of colonization and the lack of empowerment of Māori within this system. Fundamental to the problem of overrepresentation are differences between Māori approaches to justice and the structure of NZ’s criminal justice system. Through extensive consultation with Māori, Jackson (1988) outlined how formal justice approaches were individualized, punitive and adversarial, and institutionally racist. By contrast, Māori approaches to justice are less adversarial and intend to reintegrate to restore social bonds; Māori view criminal responsibility as a collective responsibility (of whānau, or family; Pratt, 1992; Tauri, 2005). Tauri (1999) suggested that biculturalization in Aotearoa NZ has resulted in a criminal justice system that superficially recognizes Māori knowledge and expertise without true empowerment. Webb (2017) argued criminal justice responses have been largely ineffective toward reducing Māori offending due to these fundamental cultural differences and lack of empowerment, alongside other effects of colonization (e.g., social marginalization, see Cunneen & Tauri, 2019).

Although risk assessment studies in Australia and Canada typically include a small proportion of Indigenous people, Māori are often the largest (or close to the largest) ethnicity group in NZ studies (e.g., Davies et al., 2022; Lloyd, Hanson et al., 2020; Yesberg & Polaschek, 2015). Despite this, we are unaware of publicly available, peer-reviewed empirical research that specifically validates risk tools with Māori samples or compares tool performance between Māori and other subgroups, such as NZ European people. Similar to legal cases in Australia (*Director of Public Prosecutions for Western Australia v. Samson*, 2014) and Canada (*Ewert v. Canada*, 2015), in 2002, an NZ probation officer and Ngāti Kahungunu (the third-largest NZ iwi/tribe) brought before the Waitangi Tribunal^2^ a claim that two risk tools developed by NZ Department of Corrections disadvantaged Māori (Waitangi Tribunal, 2005). The Tribunal concluded that the claimants were not disadvantaged by one tool used with both Māori and non-Māori, the Risk of ReConviction*Risk of ReImprisonment (RoC*RoI; Bakker et al., 1999). However, due to lack of empirical research, the Tribunal concluded they could not determine the possibility of disadvantage due to the second, culture-specific risk assessment, Māori Culture Related Needs (MaCRNs; McFarlane-Nathan, 1999). Subsequently, NZ Department of Corrections discontinued MaCRNs after completing an unpublished evaluation (as reported by Morrison, 2009).

In addition to internally developed tools, NZ Department of Corrections uses tools developed by researchers outside Aotearoa NZ (Tamatea, 2017). One is Dynamic Risk Assessment for Offender Re-entry (DRAOR; Serin, 2007), a case management tool designed for community corrections settings that was developed in Canada but originally validated in NZ. Nationally, community corrections case managers rate and reassess the dynamic risk factors (Stable subscale), destabilizing factors (Acute subscale), and strength factors (Protect subscale) within DRAOR. Stable risk factors can gradually change over time (e.g., problems with interpersonal attachment with others or sense of entitlement). Acute factors may change rapidly (e.g., substance use or unemployment). Based on extant research, items on Protect are likely promotive factors (i.e., on the same dimension as cognitive and social risk factors; Lloyd, Perley-Robertson, & Serin, 2020; Yesberg, 2015).

Corrections agencies in other countries use DRAOR (e.g., see Chadwick, 2020; K. Singh & Samion, 2016), but most current DRAOR research originated from Aotearoa NZ and, thus, these samples contain substantive Māori participants (e.g., see Lloyd, Hanson et al., 2020; Polaschek & Yesberg, 2017; Scanlan et al., 2020; Yesberg & Polaschek, 2015; Yesberg et al., 2015). Using Rice and Harris’s (2005) interpretation of AUC values (and the closely similar statistic, the *c*-index; L. M. Helmus & Babchishin, 2017) of .56, .64, and .71 as small, moderate, and large effects, respectively, evaluations of DRAOR subscale scores demonstrated small to moderate discrimination for general recidivism (AUCs = .57–.70) within the general parole population (Lloyd, Hanson et al., 2020), high-risk males on parole (Davies et al., 2022; Yesberg & Polaschek, 2015), and females on community supervision sentences (Scanlan et al., 2020). Study methodologies have differed by focusing on either one initial assessment or including reassessments into the model. Discrimination was strongest using reassessments to predict general recidivism (including breaches) with a general parole population (Stable *c*-index = .64, Acute *c*-index = .65, Protect *c*-index =.63; Lloyd, Hanson et al., 2020). Two studies have analyzed measurement invariance across time (Davies et al., 2022; Lloyd, Hanson et al., 2020). Both studies demonstrated invariance comparing ratings from prison release to ratings approximately 3 and 6 months following release. Despite the Aotearoa NZ research samples comprising 42.5% to 64.5% Māori (including the initial validation sample; Lloyd, Hanson et al., 2020), we are not aware of any peer-reviewed research that has evaluated DRAOR’s psychometric properties across Māori and NZ European subsamples.

## The Present Study

NZ Department of Corrections is committed to addressing overrepresentation of Māori under their care (NZ Department of Corrections, 2019). We agree with Tamatea (2017) that explicit evaluation of potential bias within risk tools is an important and necessary element toward this goal, even if evaluation is clearly not sufficient alone. Using a data set provided by NZ Department of Corrections, we assessed whether DRAOR scores demonstrated measurement and prediction invariance across NZ Māori^3^ and NZ European subsamples. Because there was little prior research to guide us, our research was exploratory and addressed the following research questions:

**Research Question 1:** Does the latent factor structure underlying DRAOR scores differ between Māori and NZ European subsamples?
**Research Question 2:** Do DRAOR scores discriminate recidivists versus nonrecidivists to the same degree among Māori and NZ European subsamples?
**Research Question 3:** Are the predicted absolute recidivism rates associated with each DRAOR subscale score equivalent across Māori and NZ European subsamples?

## Method

### Transparency and Openness

NZ Department of Corrections owns the data described in this article; we used these data with their permission and can share the data only with written permission from NZ Department of Corrections. Analysis code for this study is available by emailing the corresponding author. This study was not preregistered; it is a fully exploratory attempt to observe associations within routine field-generated data. Our conclusions are not definitive, and our results require replication. We report how we determined our sample size, all data exclusions, all manipulations, and all measures in the study.

### Participants

We analyzed a subsample from a data set previously used to explore other research questions about predictive validity (de Roos et al., 2022; Lloyd, Hanson et al., 2020; Lloyd, Perley-Robertson, & Serin, 2020; Stone et al., 2021, 2022, 2023); the full data set contains records for all people (*N* = 3,694) released from prison on parole in Aotearoa NZ between April 1, 2010, and March 31, 2012. For our present analyses, we removed 273 records with missing data (either release date or DRAOR assessment information; see Lloyd, Hanson et al., 2020 for further information). Furthermore, we retained only people (*n* = 3,023) whose recorded ethnicity was either Māori or NZ European, omitting people (*n* = 398) recorded with Asian, non-NZ European, Pacific Islander, or other ethnicities. This simplified and, thus, enhanced model interpretation. No DRAOR assessments had missing item scores. The data set did not contain information about case managers. Our calibration analyses required a fixed follow-up period of 2 years; for these analyses, we used the subsample (*n* = 372) of all individuals who had 2 years of follow-up data from time of release.

Table 1 provides descriptive statistics for the full sample (*N* = 3,023) split by ethnicity. Among those included in our analyses, records listed 2,794 (92.4%) people as male, 226 (7.5%) as female, and three records were missing gender information. Records identified 1,812 (59.9%) Māori and 1,211 (40.1%) NZ European people. Age ranged from 17 to 86 (*M* = 34.9, *SD* = 11.5) years. Most participants were serving sentences for nonsexual violent (43.9%), nonviolent (39.0%), or sexual violent (15.8%) convictions, with the remaining convictions for breaches of parole conditions or driving-related convictions (1.3%). The 2-year fixed follow-up sample (*n* = 372) was primarily male (96.2%; no missing records), with records listing 217 (58.3%) people as Māori and 155 (41.7%) people as NZ European. Age ranged from 17 to 86 (*M* = 35.7, *SD* = 11.1) years. Convictions were for nonsexual violent (46.2%), nonviolent (36%), sexual violent (15.9%), or driving-related (1.9%) convictions. Table 2 presents descriptive statistics for the subsample defined as having 2 full years of follow-up.

**Table 1:** Descriptive Statistics of Full Sample Participants’ Baseline Age, DRAOR Subscale Scores, Recorded Gender, Index Offense, and General Recidivism Split by Ethnicity.

Demographic/measure	NZ European (*n* = 1,211)					NZ Māori (*n* = 1,812)					Total sample (*N* = 3,023)				
	*n*	%	Range	*M*	*SD*	*n*	%	Range	*M*	*SD*	*n*	%	Range	*M*	*SD*
Age	1,210	—	17 to 86	37.40	12.62	1,811	—	17 to 80	33.24	10.39	3,021	—	17 to 86	34.91	11.51
Days between release and baseline DRAOR	1,211	—	−30 to 28	1.86	4.55	1,812	—	−30 to 28	1.96	4.97	3,023	—	−30 to 28	1.92	4.80
DRAOR scores															
Stable	1,211	—	0 to 12	5.77	2.50	1,812	—	0 to 12	6.76	2.47	3,023	—	0 to 12	6.36	2.53
Acute	1,211	—	0 to 14	5.55	2.33	1,812	—	0 to 14	6.19	2.41	3,023	—	0 to 14	5.93	2.40
Protect	1,211	—	0 to 12	6.46	2.41	1,812	—	0 to 12	5.75	2.31	3,023	—	0 to 12	6.03	2.38
Gender															
Male	1,122	92.65	—	—	—	1,672	92.27	—	—	—	2,794	7.48	—	—	—
Female	89	7.35	—	—	—	137	7.56	—	—	—	226	92.42	—	—	—
Index Offense															
Non-sexually violent	396	32.70	—	—	—	932	51.46	—	—	—	1,328	43.93	—	—	—
Non-violent	548	45.25	—	—	—	630	34.79	—	—	—	1,178	38.97	—	—	—
Sexually violent	253	20.89	—	—	—	224	12.37	—	—	—	477	15.78	—	—	—
Driving / breaches	14	1.16	—	—	—	25	1.38	—	—	—	39	1.26	—	—	—
Recidivism events	262	21.64	—	—	—	571	31.51	—	—	—	833	27.56	—	—	—

*Note.* Ethnicity was dichotomously coded indicating NZ European (0) or NZ Māori (1). DRAOR = Dynamic Risk Assessment for Offender Re-entry (Serin, 2007); NZ = New Zealand; Index Offense = most serious charge that led to imprisonment immediately prior to baseline measures.

**Table 2:** Descriptive Statistics of 2-Year Fixed Follow-Up Subsample’s Baseline Age, DRAOR Subscale Scores, Recorded Gender, Index Offense, and General Recidivism Split by Ethnicity.

Demographic/measure	NZ European (*n* = 155)					NZ Māori (*n* = 217)					Total sample (*N* = 372)				
	*n*	%	Range	*M*	*SD*	*n*	%	Range	*M*	*SD*	*n*	%	Range	*M*	*SD*
Age	155	—	19 to 86	37.83	12.50	217	—	17 to 61	34.12	9.81	372		17 to 86	35.67	11.15
Days between release and baseline DRAOR	155	—	−5 to 22	3.57	4.73	217	—	−30 to 23	3.56	6.76	372		−30 to 23	3.68	5.99
DRAOR scores															
Stable	155	—	0 to 12	5.63	2.65	217	—	0 to 12	6.48	2.77	372		0 to 12	6.13	2.75
Acute	155	—	0 to 13	5.87	2.71	217	—	0 to 13	6.71	2.66	372		0 to 13	6.36	2.71
Protect	155	—	0 to 12	6.48	2.72	217	—	0 to 12	5.86	2.67	372		0 to 12	6.12	2.71
Gender															
Male	150	96.77	—	—	—	208	95.85	—	—	—	358	96.24			
Female	5	3.23	—	—	—	9	4.15	—	—	—	14	3.76			
Index offense															
Nonsexually violent	55	35.48	—	—	—	117	53.92	—	—	—	172	46.23	—	—	—
Nonviolent	66	42.58	—	—	—	68	31.34	—	—	—	134	36.02	—	—	—
Sexually violent	30	19.35	—	—	—	29	13.36	—	—	—	59	15.86	—	—	—
Driving/breaches	4	2.58	—	—	—	3	1.38	—	—	—	7	1.88	—	—	—
Recidivism events	60	38.71	—	—	—	111	48.84	—	—	—	171	45.97	—	—	—

*Note.* Ethnicity was dichotomously coded indicating NZ European (0) or NZ Māori (1). DRAOR = Dynamic Risk Assessment for Offender Re-entry (Serin, 2007); NZ = New Zealand; Index Offense = most serious charge that led to imprisonment immediately prior to baseline measures.

Independent-samples *t* tests and chi-square tests of independence indicated that the subsample with 2 full years follow-up (*n* = 372) differed on few variables from the subsample with less than 2 years follow-up (i.e., *n* = 2,651). There was a greater average time between prison release and initial DRAOR assessment for Māori with 2 full years follow-up (*M* = 3.76, *SD* = 6.75) than Māori with less than 2 years follow-up (*M* = 1.72, *SD* = 4.63), *t*(244.35) = −4.31, *p* < .001, *g* = −.41. This effect also occurred comparing NZ European with (*M* = 3.57, *SD* = 4.73) and without 2 full years follow-up (*M* = 1.61, *SD* = 4.46), *t*(196.32) = −4.85, *p* < .001, *g* = −.44. There was a greater proportion of males in the sample of NZ Europeans with 2 full years follow-up (96.8%) than NZ Europeans with less than 2 years follow-up (92.0%), χ^2^(1) = 4.44, *p* = .04. Māori with 2 full years follow-up (*M* = 6.71, *SD* = 2.66) had a higher average DRAOR Acute scores than Māori with less than 2 years follow-up (*M* = 6.12, *SD* = 2.37), *t*(264.72) = 3.08, *p* = .002, *g* = −.24, with no other observed differences on DRAOR scores. Due to longer observation, a greater proportion of people had recorded recidivism when followed a full 2 years (Māori = 51.2%; NZ European = 38.7%) compared with those with less than 2 years follow-up: Māori = 28.8%, χ^2^(1) = 44.06, *p* < .001; NZ European = 19.1%, χ^2^(1) = 30.57, *p* < .001.

### Measures

#### Dynamic Risk Assessment for Offender Re-Entry

DRAOR (Serin, 2007) is a 19-item case management tool designed for case managers to assess clients under correctional supervision in the community, with items drawn from the body of empirical knowledge about crime prediction and desistance from crime (see Bonta & Andrews, 2017; Maruna, 2001). The tool contains three conceptually distinct subscales. The Stable subscale (six items; peer associations, attitudes toward authority, impulse control, problem-solving, sense of entitlement, and attachment with others) assesses risk factors that, although dynamic, are more enduring than the Acute items. The Acute subscale (seven items; substance abuse, anger/hostility, opportunity/access to victims, negative mood, employment, interpersonal relationships, and living situation) contains dynamic risk and destabilizing factors that can change rapidly. The Protect subscale (six items; responsive to advice, prosocial identity, realistic high expectations, costs/benefits supportive of staying crime-free, social support, and social control) assesses strength factors related to lower likelihood of recidivism or desistance from crime. Scores on Stable and Acute items are *not a problem* (0), *slight or possible problem* (1), and *definite problem* (2), with possible scores ranging 0 to 12 and 0 to 14, respectively. Scores on Protect are *not an asset* (0), *slight or possible asset* (1), and *definite asset* (2), with possible scores ranging 0 to 12. NZ Department of Corrections provided case managers with standardized training on scoring procedures; then, corrections supervisors maintained regular oversight and quality assurance.

The three DRAOR scales categorize 19 constructs by temporal and directional relationship to recidivism. The intention is that each item assesses one construct; therefore, the intention is not for the DRAOR scales to represent unidimensional latent constructs. More comprehensive assessment of numerous risk-relevant constructs maximizes prediction and prevention efforts while maintaining the practicality of risk tools (Babchishin & Hanson, 2020). As such, resulting scores measure a propensity for future criminal activity, and factors obtained through factor analyses are propensities to behaviors, not conceptual constructs.

#### Recidivism

We defined recidivism as any new criminal conviction that occurred within the date range provided in the database (April 1, 2010, to July 18, 2012), excluding convictions for breaches of supervision orders. For our calibration analyses, we fixed the follow-up timeframe to 2 years.

#### Demographic Information

We drew participant gender, age, criminal history, and classification as Māori or NZ European from official records provided by NZ Department of Corrections. At the time of data entry, Māori and NZ European status may or may not have been self-identified.

### Procedure

#### Plan of Analysis

We obtained ethics approval to use these data from institutional review boards at the following institutions: Swinburne University of Technology and Carleton University. Although the database contained repeated DRAOR assessments, we confined our analyses to DRAOR assessments that occurred closest to the day of prison release. When DRAOR assessments did not occur on the day of release, we selected a prerelease DRAOR assessment (typically within the week prior to release, up to a maximum of 30 days before release). When prerelease assessments did not occur, we selected the first postrelease assessment (typically within 7 days of release, up to no more than a maximum of 28 days). Table 1 provides descriptive statistics by subsample.

#### Measurement Invariance

To investigate whether the underlying factor structure of DRAOR scores was invariant across Māori and NZ European groups, we used multigroup exploratory structural equation modeling (ESEM; Asparouhov & Muthén, 2009) analyzed within Mplus (Version 8.4; L. K. Muthén & Muthén, 2017). We used this exploratory approach to assess multigroup invariance because DRAOR subscales are not structured to represent latent constructs. We did not have a theoretical basis for confirming prespecified latent constructs. ESEM is also robust to violations of unidimensionality (Marsh et al., 2013). ESEM requires calculating and comparing two models. The first model, commonly referred to as the noninvariant or configural model, allowed factor loadings and item thresholds to vary across groups. Following van de Schoot et al.’s (2012) guidelines, configural model fit was adequate if the comparative fit index (CFI) and Tucker–Lewis index (TLI) were > .90 (>.95 would be optimal) and root mean square error of approximation (RMSEA) was <.08 (<.05 would be optimal). The second model was the scalar (or strong) invariance model that constrains both factor loadings and item thresholds to be equal across groups. When the scalar model shows equivalent, or improved, fit compared with the configural model, this demonstrates scalar invariance, allowing researchers to conclude that raters scored items similarly across groups. It is common in tests of measurement invariance to constrain factor loadings separately during an interim step, still allowing thresholds to vary across groups (the metric model), but the metric model is not appropriate when using ESEM, nor is it an option within Mplus (B. O. Muthén, 2013).

Following previous studies (Chadwick, 2020; Davies et al., 2022; Lloyd, Hanson et al., 2020), we used a three-factor structure for DRAOR within the ESEM analyses. For ordinal categorical data with less than five categories, weighted least squares estimation methods with robust corrections are more appropriate than maximum likelihood methods (see Sellbom & Tellegen, 2019). Because DRAOR item scoring is ordinal and each item has three categories, we used a robust weighted least squares estimation method with a variance-adjusted chi-square test statistic (WLSMV), Geomin oblique factor rotation, Theta estimation, and polychoric correlations. It is typical for researchers to use change in approximate fit indices to assess whether the scalar model shows equivalent or improved fit over the configural model. For example, many rely on Chen’s (2007) guidelines for change in approximate fit indices (i.e., CFI, RMSEA, standardized root mean square residual), but Chen did not develop these guidelines with ordinal data and did not use WLSMV estimation. Subsequent research concluded that change in approximate fit indices is an inappropriate measure of invariance when using WLSMV (Sass et al., 2014). Instead, the chi-square difference test (the DIFFTEST result in Mplus) is currently the only appropriate criterion (L. K. Muthén, personal communication, June 15, 2020), even though this test is overly sensitive when sample sizes are large (Browne et al., 2002). Thus, a statistically nonsignificant chi-square difference test (*p* > .05) when comparing the configural and scalar models indicates scalar invariance.

#### Discrimination

To evaluate whether DRAOR scores discriminate recidivists from nonrecidivists equivalently within Māori and NZ European subsamples, we conducted a three-step analysis. First, in Models 1, 4, and 7, for each DRAOR subscale (Stable, Acute, and Protect respectively), we used traditional Cox regression survival analyses (see Singer & Willet, 2003) to determine whether subscale scores discriminated between recidivists and nonrecidivists within the full sample. This model accounts for follow-up times that vary across individuals. Second, in Models 2, 5, and 8, we extended Models 1, 4, and 7, by using multilevel Cox regression (for an introduction to this model, see Austin, 2017; Hox, 2010) by nesting participants within subgroups (Māori and NZ European); this model allows recidivism base rates to vary across subgroups. Finally, in Models 3, 6, and 9, we further extended the multilevel Cox regression models by additionally allowing DRAOR discrimination validity to vary by subgroup. We compared differences across model fit using chi-square statistics; a statistically significant improvement in model fit in the final step indicates that discrimination validity differed across subgroup. This analysis is functionally equivalent to testing a discrimination-by-subgroup interaction. This resulted in nine models (with three models for each DRAOR subscale). We conducted discrimination analyses using Coxph (Therneau, 2020a) and Coxme (Therneau, 2020b) packages in R (Version 3.6).

For each Cox regression model, we calculated Akaike’s information criterion (AIC; Akaike, 1974), Bayesian information criterion (BIC; Schwarz, 1978), and a weighted *c*-index (Heagerty & Zheng, 2005). Both AIC and BIC are measures of model fit based on deviance that include penalties based on number of parameters. Differences in AIC/BIC values across models are meaningful with lower values indicating improved model fit (Burnham & Anderson, 2004). Heagerty and Zheng’s (2005) weighted average *c*-index represents the probability that a randomly selected person who recidivated had a higher DRAOR score than a randomly selected person who did not recidivate, taking follow-up time into account. The *c*-index and traditional AUCs share similar interpretations. Small, moderate, and large effects are associated with scores of .56. .64, and .71, respectively (L. M. Helmus & Babchishin, 2017).

#### Calibration

For the calibration analyses, we truncated the sample to create a fixed 2-year follow-up. Using binary logistic regression in R, we first derived predicted absolute recidivism rates for each score on each subscale by subgroup. Typically (for example, see Gonçalves et al., 2020; Gregório Hertz et al., 2021; Leguízamo et al., 2017; Olver et al., 2021), researchers calculate expected/observed (E/O), or predicted/expected indices (Hanson, 2017). These indices are an effect size representing the difference between (a) the number of observed (or predicted, if derived through logistic regression) recidivists and (b) the number of expected (usually derived from tool norms) recidivists. This process determines sample calibration to the tool’s norms. However, currently, DRAOR does not have comparison norms and, instead, our present consideration is equality across subgroups. It is possible to calculate E/O indices to compare groups without norms (see Wardrop, 2020), but this requires aggregating extreme scores. We believe this is not ideal, so we calculated the average marginal effect of subgroup at each score. Using the Margins package (Leeper et al., 2018) in R, we determined change in predicted absolute recidivism rates at each possible subscale score, 95% confidence intervals, and statistical significance levels associated with each difference. Change in predicted absolute recidivism rates can be interpreted directly as the magnitude of the effect. For an introduction to this approach, see Long and Mustillo (2021).

## Results

### Exploratory Structural Equation Modeling

The initial ESEM configural model demonstrated acceptable fit, χ^2^(234, *N* = 3,023) = 1,864.11, *p* < .0001, CFI = .960, TLI = .941, RMSEA = .068. Tables S1 and S2 in the Online Supplemental Materials present factor loadings and thresholds from the configural model. Next, we constrained factor loadings and item thresholds to be equal across subgroups (the scalar model), resulting in scalar noninvariance, χ^2^(64, *N* = 3,023) = 130.13, *p* < .0001 (Mplus χ^2^ DIFFTEST), CFI = .969, TLI = .964, RMSEA = .053.^4^ This suggests there was unequal scoring across Māori and NZ European subgroups. We examined modification indices; these identified four DRAOR items as the potential sources of inequality, located at both thresholds within DRAOR Stable *peer associations* (i.e., the initial progression from *not a problem* to *slight/possible problem* and the next progression to *definite problem*), the second threshold within DRAOR Stable *sense of entitlement*, the second threshold within DRAOR Acute *opportunity/access to victims*, and the first threshold within DRAOR Acute *negative mood*. Table 3 displays the modification indices for these item thresholds. These threshold results indicate that, compared with NZ European people, raters more likely scored Māori as having *slight/possible problem* on antisocial *peer associations*. Raters also more likely scored Māori clients with a *definite problem* with *sense of entitlement* than NZ European people. By contrast, raters more likely scored NZ European clients with a *definite problem* for *peer associations* and *opportunity/access to victims* and a *slight/possible problem* on *negative mood* compared with Māori clients.

**Table 3:** Standardized Thresholds and Threshold Modification Indices for DRAOR Items Identified by Mplus.

DRAOR item	Scores					Threshold 1				Threshold 2			
	NZ European		NZ Māori			NZ European		NZ Māori		NZ European		NZ Māori	
	*M*	*SD*	*M*	*SD*	*d*	Estimate	MI	Estimate	MI	Estimate	MI	Estimate	MI
Stable													
Peer associations	0.99	0.60	1.23	0.57	.58***	−1.024	30.043	−1.057	30.026	0.804	24.916	0.831	24.919
Attitudes toward authority	0.75	0.65	0.94	0.64	.64***	−0.369	—	−0.376	—	1.207	—	1.230	—
Impulse control	1.12	0.59	1.29	0.55	.56***	−1.203	—	−1.270	—	0.731	—	0.772	—
Problem-solving	1.07	0.56	1.24	0.55	.55***	−1.129	—	−1.214	—	0.845	—	0.909	—
Sense of entitlement	1.02	0.62	1.12	0.62	.62***	−0.829	—	−0.824	—	0.915	16.878	0.909	16.934
Attachment with others	0.82	0.58	0.93	0.57	.57***	−0.585	—	−0.601	—	1.353	—	1.391	—
Acute													
Substance abuse	0.70	0.67	0.81	0.65	.66***	−0.239	—	−0.267	—	1.141	—	1.274	—
Anger/hostility	0.41	0.58	0.54	0.63	.61***	0.306	—	0.289	—	1.724	—	1.625	—
Opportunity/access to victims	0.83	0.59	0.94	0.59	.59***	−0.580	—	−0.636	—	1.189	10.199	1.306	10.206
Negative mood	0.47	0.58	0.48	0.60	.60	0.279	16.635	0.299	16.624	1.645	—	1.761	—
Employment	1.11	0.64	1.16	0.62	.63	−0.990	—	−1.024	—	0.673	—	0.696	—
Interpersonal relationships	1.57	0.68	1.67	0.61	.64***	−1.248	—	−1.325	—	−0.487	—	−0.518	—
Living situation	0.47	0.59	0.59	0.63	.61***	0.177	—	0.174	—	1.644	—	1.607	—
Protect													
Responsive to advice	1.08	0.50	0.99	0.49	.49***	−1.387	—	−1.437	—	0.905	—	0.938	—
Prosocial identity	1.01	0.52	0.87	0.52	.52***	−1.060	—	−1.141	—	1.061	—	1.142	—
Realistic high expectations	1.17	0.59	1.03	0.55	.57***	−1.279	—	−1.370	—	0.629	—	0.674	—
Costs/benefits of staying crime-free	1.07	0.53	0.95	0.52	.53***	−1.211	—	−1.298	—	0.881	—	0.944	—
Social support	1.20	0.58	1.08	0.57	.57***	−1.361	—	−1.372	—	0.579	—	0.584	—
Social control	0.94	0.50	0.83	0.48	.49***	−1.013	—	−1.041	—	1.336	—	1.372	—

*Note. N* = 3,023 people (NZ European = 1,211; NZ Māori = 1,812), DRAOR = Dynamic Risk Assessment for Offender Re-entry (Serin, 2007); Mplus (L. K. Muthén & Muthén, 2017); NZ = New Zealand; MI = Modification index.

*** *p* < .001.

We next retested the scalar model, but allowed the thresholds identified within these four items (as listed in Table 3) to freely vary across groups, but again observed a noninvariant model, χ^2^(59, *N* = 3,023) = 83.92, *p* = .018, CFI = .970, TLI = .965, RMSEA = .053. However, this comparison was noninvariant at the *p* < .01 level and the DIFFTEST is overly sensitive when sample sizes are large. Still, we retested both the configural, χ^2^(126, *N* = 3,023) = 1,046.48, *p* < .0001, CFI = .972, TLI = .953, RMSEA = .070, and scalar models after fully removing these four items, and we observed a statistically nonsignificant chi-square difference test, χ^2^(48, *N* = 3,023) = 60.89, *p* = .10, CFI = .980, TLI = .976, RMSEA = .050. This indicated scalar invariance for a reduced 15-item DRAOR model across Māori and NZ European individuals. In other words, in these data, if DRAOR did not include *peer associations, opportunity/access to victims, sense of entitlement*, and *negative mood*, case manager scoring would have been similar across Māori and NZ European subsamples for all remaining 15 items. We present the mean DRAOR Stable and Acute scores for this 15-item measure in Table S3 in the Online Supplemental Materials.

### Multilevel Cox Regression

During the full follow-up, official records identified recidivism (any type of new criminal conviction) for 833 (27.6%) people. We present results from three series of three Cox regression prediction models in Table 4. Across all participants (prior to nesting into subgroups), DRAOR Stable, Acute, and Protect subscales each demonstrated statistically significant discrimination for classifying people with versus without future recidivism. After nesting and allowing the model to estimate a different base rate for each subgroup (the random intercept), we observed substantially improved model fit across all three models. This reflects the known recidivism base rate difference across these subgroups. In our final step, allowing the relationship between DRAOR subscale score and recidivism to vary across subgroup did not improve model fit for any of the final models in each series of models. In other words, progressively higher DRAOR subscale scores were associated with progressively higher recidivism rates (e.g., one score difference in DRAOR Stable was associated with approximately 14% greater likelihood of recidivism) and this discrimination validity did not differ across subgroups.

**Table 4:** Multilevel Cox Regression Survival Analyses With DRAOR Subscale Scores Predicting General Recidivism and Ethnicity (Māori) as a Level 2 Grouping Variable.

Models	Fixed effects models with predictors only		Random effects models with intercept (base rate) varying across ethnicity		Random effects models with intercept and predictor effects varying across ethnicity	
	*B* (*SE*) *e*^B^ [95% CI]	*c*-index	*B* (*SE*) *e*^B^ [95% CI]	*c*-index	*B* (*SE*) *e*^B^ [95% CI]	*c*-index
Models 1–3: DRAOR Stable	0.14*** (0.014) 1.15 [1.12, 1.18]	0.59	0.13*** (0.014) 1.14 [1.11, 1.17]	0.60	0.13*** (0.014) 1.14 [1.11, 1.17]	0.60
	Random effects					
Intercept variance	—		0.051		0.051	
Predictor variance	—		—		0.00039	
	Goodness of fit					
AIC/BIC	12,671.80/12,676.52		12,654.38/12,663.83		12,655.83/12,670.01	
Δχ^2^ vs. preceding model^a^	—		15.51***		0.0076	
Models 4–6: DRAOR Acute	0.15*** (0.014) 1.16 [1.13, 1.20]	0.60	0.14*** (0.014) 1.15 [1.12, 1.18]	0.61	0.14*** (0.014) 1.15 [1.12, 1.18]	0.61
	Random effects					
Intercept variance	—		0.061		0.061	
Predictor variance	—		—		0.000049	
	Goodness of fit					
AIC/BIC	12,666.37/12,671.09		12,644.97/12,654.42		12,646.92/12,661.10	
Δχ^2^ vs. preceding model^a^	—		19.31***		0.014	
Models 7–9: DRAOR Protect	−0.13*** (0.014) 0.88 [0.86, 0.91]	0.58	−0.12*** (0.014) 0.89 [0.86, 0.91]	0.59	−0.12*** (0.015) 0.89 [0.86, 0.91]	0.60
	Random effects					
Intercept variance	—		0.067		0.074	
Predictor variance	—		—		0.0026	
	Goodness of fit					
AIC/BIC	12,701.40/12,706.12		12,677.72/12,687.17		12,676.19/12,690.37	
Δχ^2^ vs. preceding model^a^	—		21.49***		0.18	

*Note. N* = 3,023 people (NZ European = 1,211; NZ Māori = 1,812), *n* recidivism events = 833. Ethnicity was dichotomously coded indicating NZ European (0) or NZ Māori (1). Akaike information criterion (AIC) was calculated using penalized maximum likelihood function. Bayesian information criterion (BIC) was calculated using the number of events as the sample size (see Volinsky & Raftery, 2000) and penalized maximum likelihood function. DRAOR = Dynamic Risk Assessment for Offender Re-entry (Serin, 2007); *e*^B^ = Hazard ratio; CI = confidence interval; *c*-index = Concordance (see Heagerty & Zheng, 2005).

a Change in chi-square across models is a comparison of model deviance.

*** *p* < .001.

### Calibration

Within the fixed 2-year follow up, records identified recidivism for 166 (55.4%) people. In Table 5, we present predicted 2-year recidivism rates for each DRAOR subscale score by subgroup, multiplying predicted probabilities by 100 to represent percentages. Estimated recidivism rates associated with DRAOR Stable scores differed, such that Māori had estimated rates 8.3% to11.8% higher than NZ European people. Similarly, predicted rates were comparatively higher for Māori by 9.2% to 12.2% and 9.8% to 13.0% across DRAOR Acute and Protect scores, respectively. Subgroup differences in predicted recidivism rates were statistically significant (*p* < .05) at every possible subscale score.

**Table 5:** Predicted Rates of General Recidivism Within 2 Years at Each DRAOR Subscale Total Score.

DRAOR subscale	NZ European (*n* = 155)				NZ Māori (n = 217)				Difference		
	*n*	*n_recidivists_*	PR	95% CI	*n*	*n_recidivists_*	PR	95% CI	ΔPR	95% CI	*p*
Stable											
0^a^	2	0	18.8	[10.1, 27.6]	4	1	27.2	[15.6, 38.8]	8.3	[0.3, 16.4]	.043*
1	9	2	21.4	[12.8, 30.0]	5	2	30.4	[19.5, 41.3]	9.0	[0.5, 17.5]	.037*
2	11	2	24.2	[15.9, 32.4]	10	3	33.9	[23.8, 43.9]	9.7	[0.7, 18.7]	.033*
3	18	3	27.2	[19.3, 35.1]	15	5	37.5	[28.5, 46.6]	10.3	[0.9, 19.7]	.032*
4	7	2	30.5	[22.8, 38.1]	20	10	41.3	[33.2, 49.4]	10.9	[1.0, 20.7]	.030*
5	15	6	33.9	[26.3, 41.5]	17	8	45.2	[37.9, 52.5]	11.3	[1.1, 21.5]	.030*
6	46	20	37.6	[29.7, 45.4]	53	27	49.2	[42.3, 56.0]	11.6	[1.2, 22.0]	.030*
7	21	11	41.4	[32.9, 49.8]	28	10	53.1	[46.2, 60.0]	11.8	[1.1, 22.4]	.030*
8	10	3	45.3	[35.8, 54.7]	16	10	57.1	[49.6, 64.5]	11.8	[1.1, 22.5]	.031*
9	8	4	49.2	[38.6, 59.9]	11	8	60.9	[52.7, 69.2]	11.7	[1.0, 22.4]	.032*
10	3	2	53.2	[41.2, 65.2]	15	9	64.6	[55.4, 73.8]	11.4	[0.9, 22.0]	.034*
11	4	1	57.1	[43.8, 70.4]	14	12	68.2	[58.1, 78.3]	11.0	[0.7, 21.4]	.037*
12^a^	1	0	61.0	[46.5, 75.5]	9	5	71.5	[60.6, 82.4]	10.6	[0.5, 20.6]	.040*
Acute											
0^a^	4	0	20.7	[11.0, 30.5]	1	0	29.9	[17.1, 42.7]	9.2	[0.6, 17.8]	.036*
1	5	0	23.0	[13.7, 32.3]	4	1	32.8	[20.9, 44.6]	9.8	[0.9, 18.7]	.031*
2	9	2	25.4	[16.6, 34.2]	7	1	35.7	[25.0, 46.5]	10.3	[1.1, 19.6]	.028*
3	14	4	28.0	[19.7, 36.3]	14	4	38.8	[29.2, 48.4]	10.8	[1.3, 20.4]	.026*
4	16	9	30.7	[22.9, 38.6]	22	9	42.0	[33.6, 50.5]	11.3	[1.5, 21.1]	.025*
5	14	7	33.6	[26.0, 41.2]	27	15	45.3	[37.8, 52.7]	11.7	[1.5, 21.8]	.024*
6	35	10	36.6	[28.9, 44.3]	29	17	48.6	[41.7, 55.4]	11.9	[1.6, 22.3]	.024*
7	26	9	39.7	[31.5, 48.0]	31	15	51.9	[45.1, 58.6]	12.1	[1.6, 22.6]	.024*
8	13	7	42.9	[33.8, 52.1]	19	8	55.1	[47.9, 62.4]	12.2	[1.6, 22.8]	.024*
9	7	4	46.2	[35.9, 56.5]	34	21	58.4	[50.3, 66.5]	12.2	[1.5, 22.8]	.025*
10	6	2	49.5	[37.7, 61.3]	14	11	61.6	[52.4, 70.7]	12.1	[1.4, 22.7]	.026*
11	4	2	52.8	[39.5, 66.1]	10	6	64.6	[54.4, 74.9]	11.8	[1.3, 22.4]	.028*
12^a^	1	0	56.1	[41.3, 70.8]	3	1	67.6	[56.3, 78.9]	11.5	[1.1, 21.9]	.030*
13^a^	1	0	59.3	[43.2, 75.4]	2	1	70.4	[58.1, 82.7]	11.1	[0.8, 21.4]	.034*
14^a b^	0	0	62.4	[45.1, 79.8]	0	0	73.1	[60.0, 86.1]	10.6	[0.5, 20.7]	.039*
Protect											
0^a^	2	0	58.5	[43.5, 73.5]	8	5	70.4	[58.8, 82.1]	11.9	[1.7, 22.0]	.022*
1	3	0	55.1	[41.5, 68.7]	4	3	67.4	[56.7, 78.1]	12.3	[2.0, 22.7]	.019*
2	4	4	51.6	[39.4, 63.7]	11	5	64.2	[54.5, 73.9]	12.7	[2.2, 23.2]	.018*
3	9	6	48.0	[37.3, 58.8]	17	12	60.9	[52.3, 69.6]	12.9	[2.3, 23.5]	.017*
4	13	8	44.5	[35.0, 54.0]	21	13	57.5	[49.8, 65.2]	13.0	[2.4, 23.6]	.016*
5	9	2	41.0	[32.6, 49.5]	13	6	54.0	[47.0, 61.0]	13.0	[2.5, 23.5]	.016*
6	50	18	37.7	[29.8, 45.5]	65	33	50.5	[43.7, 57.2]	12.8	[2.5, 23.2]	.015*
7	11	4	34.4	[26.8, 42.0]	26	13	47.0	[39.9, 54.0]	12.5	[2.4, 22.7]	.015*
8	20	5	31.3	[23.6, 39.0]	17	8	43.4	[35.6, 51.3]	12.2	[2.3, 22.0]	.016*
9	11	5	28.3	[20.3, 36.4]	12	4	40.0	[31.2, 48.9]	11.7	[2.2, 21.2]	.016*
10	10	2	25.6	[17.0, 34.1]	12	5	36.7	[26.7, 46.6]	11.1	[2.0, 20.3]	.017*
11^a^	4	0	23.0	[14.0, 31.9]	7	3	33.5	[22.4, 44.5]	10.5	[1.7, 19.3]	.019*
12^a^	9	2	20.6	[11.2, 29.9]	4	0	30.4	[18.4, 42.3]	9.8	[1.4, 18.2]	.022*
Totals	155	56			217	110					

*Note. N* = 372 people. DRAOR = Dynamic Risk Assessment for Offender Re-entry (Serin, 2007); NZ = New Zealand; PR = predicted rate of recidivism within 2 years; CI = confidence interval.

a No recidivists received this score in one or both groups. ^b^ Sample is 0 for this score in both groups.

*** *p* < .05.

## Discussion

We evaluated three critical psychometric properties for establishing that scores from a case management tool (DRAOR) can be validly applied in a community corrections setting with both Māori and NZ European people. However, because there are known differences in recidivism base rates across these subgroups, it is impossible for any applied risk tool to demonstrate cross-group equality through both classification and calibration accuracy, making the most critical research goal to quantify how and to what degree these psychometric properties differed across groups. Specific information about score interpretation within different subgroups critically assists decision-makers and assessors to more accurately and effectively use risk tools in practice.

Results demonstrated that DRAOR’s underlying factor structure was noninvariant across Māori and NZ European people due to unequal scoring thresholds on four items. For two items (*opportunity/access to victims* and *negative mood*), scoring more readily identified NZ European people as higher risk, whereas scoring on DRAOR *sense of entitlement* more readily identified Māori as higher risk. Furthermore, on *peer associations*, there was relatively higher likelihood Māori would receive a score of *slight/possible problem*, but a higher likelihood NZ Europeans would receive a score of *definite problem.* In other cross-cultural research contexts, there are methods for identifying the source of bias detected through measurement invariance (e.g., disentangling the effects of culture and language; see Bader et al., 2021), but these methods are not possible in the present context. Thus, our proposed explanations for unequal scoring thresholds are speculatory and require further exploration. Also, because DRAOR is a field-based tool, these data are limited by not including interrater reliability information. As such, some possible but untestable explanations for scoring differences include rater bias (Venner et al., 2021), cultural misinterpretation (Shepherd & Lewis-Fernandez, 2016), or true differential exposure to these four risk factors across groups, on average.

During data collection, more than 20% of corrections staff identified as Māori (NZ Department of Corrections, 2012), but still, on average, a person who does not identify as Māori would have more likely conducted DRAOR assessments with a Māori client, creating possible opportunities for European-to-Māori cultural misinterpretation. In our study, scoring patterns on DRAOR items *sense of entitlement* and *negative mood* could be consistent with clinical misinterpretation. True group-based differences in concentration of these specific risk factors may also exist. Overrepresentation of Māori in the justice system guarantees that Māori clients are more likely to have family and friends with justice involvement. This may explain why scoring more readily placed Māori on *slight/possible problem* on *peer associations* than *not a problem*. However, this does not explain why scoring more readily placed NZ Europeans on *definite problem*. Furthermore, two family-relevant strength items (*social support* and *social control* on DRAOR Protect) had equal thresholds across subgroups in the measurement invariance model. NZ Department of Corrections (2019) places great importance on whānau (family) in rehabilitation programming for Māori, noting that whānau should be viewed as a strength, not a risk factor. Scoring identified these strength factors with the same likelihood for Māori and NZ European clients. Finally, if the criminal justice system places greater scrutiny and limits on Māori compared with NZ European people, this may explain why scoring identified NZ Europeans as greater risk on *opportunity/access to victims* and Māori as having stronger attitudes of entitlement if, for example, scoring reflected demands for fairer treatment.

Still, the present study design does not allow identifying sources of noninvariance, so raters should similarly remain aware of alternate interpretations of behaviors and avoid incorrectly assuming internal attributions; DRAOR raters should be particularly mindful when scoring the four items we identified as noninvariant in this study. However, a more meaningful solution and recommendation is to ensure that scoring criteria on these items are better defined and made less ambiguous. Between data collection (from 2010 to 2012) and analyses (in 2020), a revision of the DRAOR user manual was published (in 2017). In this revision, scoring criteria became better specified through clearer rating definitions and behavior exemplars at each score. Future analyses should examine data collected after 2017 to evaluate whether clearer scoring criteria are associated with stronger measurement invariance.

On balance, there was stronger support for cross-ethnicity measurement invariance, if noting that most items (15 of 19) showed scalar invariance. Yet, we used post hoc analyses to generate scalar invariance within the truncated 15-item scale; Marsh and colleagues (2018) criticized this backward elimination process to achieve partial scalar invariance. Conclusions based on post hoc analyses when no a priori theory supported the deletion/freeing of items/thresholds are problematic. Our results may not replicate in other samples; replication is certainly required.

Although we believe evidence of measurement noninvariance across groups does not necessarily invalidate results we observed related to predictive discrimination, Millsap (2007) warned that systematic decision-making errors may occur when relying on prediction results (discrimination and calibration) alone, ignoring measurement bias. Our results highlight that this warning may be relevant for many other risk tools in corrections lacking evaluations of measurement invariance across ethnicities.

DRAOR scores demonstrated equal discrimination across subgroups (i.e., equal magnitude of increasing recidivism likelihood with increasing scores), but the well-known differences in recidivism rates across subgroups clearly featured within analyses examining calibration. Prediction models that allowed the base rate to differ by subgroup demonstrated substantially improved model fit, and we observed consistently higher predicted recidivism rates for every possible DRAOR subscale among Māori compared with NZ European people. The reasons underlying these differential recidivism rates are simultaneously fully speculative (in that our data are unable to specify the potential reasons), yet myriad and long familiar. For example, to the degree that Māori, on average, experience greater disadvantage from the justice system than NZ European people due to, for example, legal code definitions, greater law enforcement surveillance, poorer legal representation, harsher sentencing, fewer or less culturally appropriate resources to support prosocial reintegration, there will be a greater likelihood of ongoing justice contact. Furthermore, our definition of recidivism in this study is not neutral (as no definitions of recidivism are, including self-report); as such, to the degree that rearrest, reconviction, and reimprisonment are justice system actions subject to their own biases, recidivism in this study is at least partially a reflection of those biased processes. In addition, historic and persistent socioeconomic marginalization creates and sustains disenfranchisement with societal expectations (including its laws) and greater relative exposure to many of the core risk factors for law violations.

Ultimately, explaining the underlying reasons for differential base rates is beyond the scope of our data or this article and we refer readers to Cunneen and Tauri (2019) for a discussion of the adverse consequences that colonialism has had on Indigenous peoples in relation to criminal justice systems. However, it is important that case managers who use DRAOR in Aotearoa NZ are aware of the practice implications related to the differential base rates. Case managers should be aware that Māori clients have relatively higher risk of continued justice contact for all the reasons described in the preceding paragraph, and that DRAOR assessments of clients’ dynamic needs were not designed to correct for this systemic difference (e.g., by including variables that explicitly represent or are proxies of race/ethnicity or explicitly measure the many underlying reasons base rates differ). Instead, case managers can best use DRAOR scores to prioritize delivering more substantial services to higher scoring clients compared with lower scoring clients, knowing these priority decisions will identify those who need relatively more services, with each threshold identifying a relatively higher-risk group to the same degree despite the subgroup. Fortunately, in practice, NZ Department of Corrections uses DRAOR to assess case management needs and informally prioritize case management services, and this study supports the conclusion that these practices are appropriate. However, specific attention to the calibration of DRAOR scores would be required prior to using DRAOR for calibration-focused decisions, such as communicating specific recidivism likelihood rates at each score or aligning the frequency of client contact to a single DRAOR score under the assumption that it represents a universally specific threshold of recidivism likelihood.

Overall, our results support taking a comprehensive approach to evaluating the applicability of correctional risk tools across ethnicities and cultural groups. In other words, accurate conclusions about tool performance across ethnicities are related to exploring various forms of invariance, including measurement invariance and prediction invariance through both discrimination and calibration. Furthermore, these psychometric properties are related to each other, and there are inherent trade-offs when recidivism base rates differ across groups. Furthermore, this study is unique in that, to our knowledge, no prior published studies (a) evaluated predictive invariance of a correctional risk tool across Māori and non-Māori samples, or (b) assessed measurement invariance across different Indigenous and White samples using DRAOR, as the only available similar studies focused on the Psychopathy Checklist family of tools and the YLS/CMI.

### Limitations and Future Directions

Because data collection occurred in the field as part of routine practice, our analyses have some clear advantages (e.g., they can inform real-world decision-making) and disadvantages (e.g., unknown information about interrater reliability, raters, and construct validity). Although dynamic factors change over time, we used only the initial DRAOR assessments from the data set in our analyses. Prior research concluded that the latent factors underlying DRAOR scores are invariant across time (Davies et al., 2022; Lloyd, Hanson et al., 2020), so we expect that our conclusions about measurement invariance across ethnicity would similarly remain stable across time. However, this requires further research.

The most immediate outcome of DRAOR assessments is the case management strategy that the supervision officer uses to address their clients’ risk and needs. While in the present study we used recidivism as an appropriate outcome variable, we did not have access to strategies or interventions decided on by supervision officers. Therefore, our data do not allow us to make statements regarding how the relationship of DRAOR scores and case management strategies may or may not vary across ethnicity.

When assessing measurement invariance, we did not match groups on other characteristics available in the data (such as age and previous index offense). Prior studies used matching to ensure noninvariance is attributable to cross-cultural biases (see Bader et al., 2021; Han et al., 2019). However, it may simply obscure true group differences to match samples on risk-relevant characteristics that may also differ across groups due to systemic biases in the criminal justice system (e.g., more affluent defendants may more easily plea bargain criminal charges to less serious convictions). We gave preference to retaining the full sample (that represented the entire parole population at the time) over a matching procedure.

The methodology we used for our calibration analyses allows comparison of tail-end scores, whereas other approaches to calibration across subsamples are comparatively more limited. Still, we extrapolated predicted recidivism rates to scores where either no person or no recidivist received that score in our sample. Therefore, caution is warranted, and replication is required.

### Constraints on Generality

The nature of this study’s goals ties its conclusions explicitly to specific groups of people (i.e., those who identify as Māori or NZ European) in a specific country (i.e., Aotearoa NZ) who have also experienced a specific disadvantage (i.e., were incarcerated for a criminal conviction). We hope that readers will consider our set of methodologies instructive for examining cross-ethnicity psychometric invariance in other contexts, but, of course, we do not encourage applying our specific conclusions to other locations, ethnic groups, or contexts, even if using the same risk tool (i.e., DRAOR).

Furthermore, we wrote this article as researchers wanting to contribute responsibly to the integrity of the knowledgebase that supports correctional agencies using DRAOR as part of their delivery of humane and positive services to justice-involved clients. We cannot and do not make claims to speak for or tell the stories of people who identify as Māori, and we acknowledge that our status as outsiders who do not share the personal, firsthand experiences of Māori constrains the breadth of perspective we bring to this research, in ways that we may be both aware and unaware. It is our intention for this article to provide helpful information that gives new specificity to the highly problematic overrepresentation of Māori in the justice system. We hope this research can contribute toward solutions. We strongly reject any potential application of our research that would (a) undermine self-determination among Māori; (b) implement correctional practices that are not humane, client-focused, and strength-building; or (c) perpetuate existing inequalities. Please also see the Online Supplemental Material where we have written a fuller statement.

### Conclusion

Prior to implementing decision criteria with Indigenous peoples, evaluation of correctional risk assessment tools should, at a minimum, first examine measurement invariance, predictive discrimination, and prediction calibration. Structured tools are advantageous because these psychometric properties can be tested, whereas unstructured decision-making obscures these concerns. When groups have different recidivism base rates, evaluation should involve considering the inherent trade-offs that occur. Within international correctional practice, it is concerning and increasingly tenuous that assessors often apply risk tools with Indigenous clients without first identifying whether (or, more accurately, in which ways) assessment information must be interpreted differently across subgroups.

## Supplemental Material

sj-pdf-1-asm-10.1177_10731911231153838 – Supplemental material for Psychometric Properties of a Risk Tool Across Indigenous Māori and European Samples in Aotearoa New Zealand: Measurement Invariance, Discrimination, and Calibration for Predicting Criminal Recidivism Click here for additional data file. Supplemental material, sj-pdf-1-asm-10.1177_10731911231153838 for Psychometric Properties of a Risk Tool Across Indigenous Māori and European Samples in Aotearoa New Zealand: Measurement Invariance, Discrimination, and Calibration for Predicting Criminal Recidivism by Darcy J. Coulter, Caleb D. Lloyd and Ralph C. Serin in Assessment

## Notes

1. We use the term *Indigenous* to refer to numerous and diverse groups of people from Australia, Aotearoa New Zealand, and Canada. We respect and acknowledge that this term is not preferred by all peoples described in this article, and that all individuals and groups are certainly not analogous to one another, including when they reside or originate from within the boundaries of the same present-day country.

2. The Waitangi Tribunal is a standing commission of inquiry that makes recommendations following claims bought by Māori related to breaches of promises under the Treaty of Waitangi/Te Tiriti o Waitangi, an agreement that establishes, guides, and governs the relationship between the Aotearoa New Zealand government and Māori.

3. We refer to New Zealand Māori because our sample excluded Cook Islands Māori. Hereafter, we use “Māori” to refer to New Zealand Māori only.

4. The unexpected direction of the change in fit indices across models highlights that change in approximate fit indices is an inappropriate measure of invariance when using WLSMV (Sass et al., 2014).
