# Supplementary material for: Psychometric Properties of a Risk Tool Across Indigenous Māori and European Samples in Aotearoa New Zealand: Measurement Invariance, Discrimination, and Calibration for Predicting Criminal Recidivism
Source: Assessment. 2023 Feb 22;30(8):2560–79. doi: 10.1177/10731911231153838 (PMC10655698; doi:10.1177/10731911231153838)
Supplement: sj-pdf-1-asm-10.1177_10731911231153838 – Supplemental material for Psychometric Properties of a Risk Tool Across Indigenous Māori and European Samples in Aotearoa New Zealand: Measurement Invariance, Discrimination, and Calibration for Predicting Criminal Recidivism [file sj-pdf-1-asm-10.1177_10731911231153838.pdf]

Version 2 – Uploaded on 10 March 2023
Online Supplemental Materials
Statement from the Authors

Dear readers,

We write to you from Melbourne, Australia, located on the traditional lands of the Wurundjeri peoples, and Ottawa, Canada, located on the traditional lands of the Algonquin Anishnaabeg peoples. We recognize and acknowledge these Indigenous peoples' long-standing connection and contribution to these lands as their custodians and stewards. We pay our respects to their leaders, past and present, and extend that respect to any Indigenous readers. We acknowledge that Indigenous sovereignty has not been ceded.

We have written an article describing our analysis of ratings recorded by corrections staff employed by Ara Poutama Aotearoa (New Zealand Department of Corrections) as they provided supervision services to Māori people in Aotearoa New Zealand. We identify our ethnicity as European Caucasian, and our ancestry as descendants of settlers and immigrants who arrived in countries with established peoples. We do not identify as Māori. It is clear to us that we cannot represent and it is not our intention that our words would represent the views of Māori people nor communicate the stories of Māori people. Our article is written from our personal viewpoints, formed by our own ethnic identities and experiences of the world, shaped by our advantages and the sum of the systemic treatment we have characteristically received from others and societal institutions throughout our lives. We are outsiders to Aotearoa New Zealand, and our viewpoints similarly do not represent the perspectives of NZ Europeans nor staff at Ara Poutama Aotearoa (New Zealand Department of Corrections).

We wrote this article because we regularly contribute to the knowledgebase about a correctional case management tool, the Dynamic Risk Assessment for Offender Re-entry (DRAOR), thereby supporting its use by corrections agencies. We write about DRAOR in ways that both promote evidence of its helpful features and clarify its limitations. From this stance, we believed it was inappropriate to turn a blind eye to any potential limitations related to cross-ethnicity validity, when we had the opportunity to examine these limitations through our access to DRAOR data.

We appreciate that our voice in the broader and important conversation about overrepresentation of Indigenous peoples within international justice systems is limited and can only ever offer a narrow contribution, given our lack of first-hand perspective. However, we offer this article in the hope that it may contribute to positive solutions for better understanding and reducing this clear problem of overrepresentation. Perhaps you will perceive our intentions as naïve, but (as researchers) we are prone to believing that when we generate specific information about problems, this assists the pursuit of solutions.

For our readers who identify as Māori, we hope that this article can be useful, providing specific information that might guide specific self-advocating and self-determining choices and actions. We also acknowledge the helpful transparency of Ara Poutama Aotearoa (New

Zealand Department of Corrections), both in providing these data for our analysis and regularly publishing information that identifies the nature of the problem of Māori overrepresentation. This is atypical, and it greatly enhanced our learning and data interpretation to be able to easily find publicly available information about group recidivism base rates, for example.

As we wrote in the article, we strongly reject and disavow any potential application of our research that would (1) undermine self-determination among Māori people, (2) implement correctional practices that are not humane, client-focused, and strength-building, or (3) perpetuate existing inequalities. More broadly, we believe the goals of Māori autonomy, equality, and positive experiences when engaging in correctional matters and processes cannot be achieved unless any implemented practice is transparent and repeatedly subjected to rigorous evaluation. We view our article as one small piece of this transparent evaluation process and invite any communication or criticisms of our work that will help advance these goals.

With our sincerity,

Darcy J. Coulter, Caleb D. Lloyd, & Ralph C. Serin
Melbourne, Australia & Ottawa, Canada

Table S1

Statistically Significant Standardized Factor Loadings Resulting From a Three-Factor Configural Exploratory Structural Equation Model of DRAOR Items with a Sample of NZ European and Māori People on Parole

DRAOR item	NZ European[a]						NZ Māori[b]					
	Factor 1		Factor 2		Factor 3		Factor 1		Factor 2		Factor 3	
	Λ	SE	Λ	SE	Λ	SE	Λ	SE	Λ	SE	Λ	SE
Stable												
Peer associations	**.368**	.05	*.062*	.05	-.215	.05	**.408**	.04	.188	.04	*-.081*	.05
Attitudes towards authority	**.412**	.04	.318	.06	*-.180*	.07	**.490**	.04	.396	.03	*-.057*	.05
Impulse control	**.883**	.04	*-.005*	.01	*.082*	.05	**.906**	.03	*-.031*	.03	*.037*	.04
Problem-solving	**.909**	.04	*-.126*	.06	*.008*	.01	**.860**	.03	*-.004*	.01	*-.019*	.03
Sense of entitlement	**.474**	.04	.232	.05	*-.191*	.06	**.646**	.03	.322	.03	*.048*	.04
Attachment with others	**.267**	.05	.248	.06	-.264	.06	**.396**	.04	.318	.03	*-.106*	.04
Acute												
Substance abuse	*.153*	.05	**.314**	.05	*-.018*	.04	.151	.04	**.476**	.03	*.050*	.04
Anger/hostility	*.021*	.05	**.855**	.09	*.052*	.10	*-.034*	.04	**.847**	.03	*.009*	.02
Opportunity/access to victims	.213	.05	**.282**	.06	*-.063*	.06	.282	.04	**.336**	.03	*-.051*	.04
Negative mood	*-.002*	.04	**.657**	.05	*.023*	.06	*-.009*	.02	**.731**	.03	*.013*	.03
Employment	*.160*	.05	**.194**	.06	*-.121*	.05	*.128*	.04	**.213**	.04	-.141	.04
Interpersonal relationships	**.284**	.06	*-.016*	.06	*-.029*	.06	**.181**	.05	*.057*	.04	*-.053*	.05
Living situation	*-.008*	.03	.306	.05	**-.324**	.06	*.028*	.04	**.347**	.03	-.240	.04
Protect												
Responsive to advice	*-.211*	.06	*-.03*	.04	**.615**	.05	*-.177*	.04	*-.044*	.03	**.638**	.03
Prosocial identity	*-.007*	.04	*.019*	.04	**.860**	.04	*-.060*	.03	*-.001*	.01	**.837**	.02
Realistic high expectations	*.070*	.06	*.012*	.03	**.883**	.04	*.005*	.02	*-.015*	.02	**.827**	.02
Costs/benefits of staying crime-free	*-.133*	.06	.162	.04	**.803**	.04	-.133	.03	*.061*	.03	**.760**	.03
Social support	*.115*	.06	*-.131*	.05	**.723**	.05	*.063*	.03	-.136	.03	**.647**	.03
Social control	*-.052*	.06	*.000*	.03	**.782**	.04	*.007*	.02	*.029*	.03	**.821**	.02

Note. Italicized values indicate p >.001. Bolded values indicate strongest loading for each item. DRAOR = Dynamic Risk Assessment for Offender Re-entry (Serin, 2007); NZ = New Zealand; Λ = Standardized factor loading estimate; SE = Standard error.

[a] n = 1,211. [b] n = 1,812.

Table S2

Standardized Threshold Estimates for DRAOR Items Resulting From a Three-Factor Configural Exploratory Structural Equation Model of DRAOR Items with a Sample of NZ European and Māori People on Parole

DRAOR Item	NZ European (*n* = 1,211)				NZ Māori (*n* = 1,812)			
	Threshold 1		Threshold 2		Threshold 1		Threshold 2	
	Estimate	SE	Estimate	SE	Estimate	SE	Estimate	SE
Stable								
Peer associations	-0.90	0.04	0.93	0.04	-1.44	0.04	0.52	0.03
Attitudes towards authority	-0.33	0.04	1.19	0.05	-0.72	0.03	0.92	0.03
Impulse control	-1.18	0.05	0.72	0.04	-1.67	0.05	0.41	0.03
Problem-solving	-1.16	0.05	0.87	0.04	-1.55	0.05	0.53	0.03
Sense of entitlement	-0.90	0.04	0.82	0.04	-1.09	0.04	0.64	0.03
Attachment with others	-0.61	0.04	1.33	0.05	-0.86	0.03	1.13	0.04
Acute								
Substance abuse	-0.19	0.04	1.17	0.05	-0.46	0.03	1.10	0.04
Anger/hostility	0.35	0.04	1.70	0.06	0.08[a]	0.03	1.46	0.04
Opportunity/access to victims	-0.61	0.04	1.28	0.05	-0.82	0.03	1.05	0.04
Negative mood	0.19	0.04	1.69	0.06	0.19	0.03	1.58	0.05
Employment	-1.01	0.04	0.62	0.04	-1.16	0.04	0.58	0.03
Interpersonal relationships	-1.23	0.05	-0.46	0.04	-1.46	0.04	-0.65	0.03
Living situation	0.22	0.04	1.63	0.06	-0.03[b]	0.03	1.44	0.04
Protect								
Responsive to advice	-1.35	0.05	0.97	0.04	-1.16	0.04	1.19	0.04
Prosocial identity	-1.12	0.05	1.08	0.05	-0.81	0.03	1.44	0.04
Realistic high expectations	-1.27	0.05	0.62	0.04	-1.10	0.04	0.96	0.04
Costs/benefits of staying crime-free	-1.22	0.05	0.92	0.04	-0.99	0.04	1.21	0.04
Social support	-1.36	0.05	0.56	0.04	-1.15	0.04	0.82	0.03
Social control	-0.99	0.04	1.30	0.05	-0.78	0.03	1.69	0.05

Note. All thresholds significant (p <.001) unless otherwise specified. DRAOR = Dynamic Risk Assessment for Offender Re-entry (Serin, 2007); NZ = New Zealand; SE = Standard error.

[a] p = .007. [b] p = .372.

Table S3

Means and Standard Deviations of DRAOR Stable and Acute Scores with Peer Associations, Sense of Entitlement, Access to Victims, and Negative Mood Removed

DRAOR Subscale	NZ European (*n* = 1,211)			NZ Māori (*n* = 1,812)			Total sample (*N* = 3,023)		
	Range	*M*	*SD*	Range	*M*	*SD*	Range	*M*	*SD*
Stable	0–8	3.75	1.74	0–8	4.41	1.72	0–8	4.14	1.75
Acute	0–8	3.79	1.51	0–8	4.18	1.5	0–8	4.02	1.52

Note. Ethnicity was dichotomously coded indicating NZ European (0) or NZ Māori (1). DRAOR = Dynamic Risk Assessment for Offender Re-entry (Serin, 2007); NZ = New Zealand.
